# A Systematic Review and Single Center Experience With Percutaneous Needle Tenotomy in Congenital Talipes Equinovarus (CTEV)

**DOI:** 10.7759/cureus.32812

**Published:** 2022-12-22

**Authors:** Mohit Dhingra, Yasmin Cazorla Bak, Fortune Edokpayi, Han Hong Chong, Srinivasan Shyamsundar

**Affiliations:** 1 Trauma and Orthopaedic Department, Kettering General Hospital, Kettering, GBR; 2 Trauma and Orthopaedic Department, University Hospitals of Leicester NHS Trust, Leicester, GBR

**Keywords:** congenital talipes equinovarus, ctev, clubfoot, needle, percutaneous, achilles tenotomy

## Abstract

Percutaneous scalpel tenotomy is frequently performed as part of congenital talipes equinovarus (CTEV) to correct the equinus deformity. The use of a scalpel is associated with complications such as neurovascular bundle damage and pseudoaneurysms. In the literature, a percutaneous large-bore needle has been found to be a safer alternative to a scalpel for performing tenotomies. The goal of this study was to conduct a systematic review and report a single-center case series on the use of percutaneous needle tenotomy in the treatment of CTEV.

A Preferred Reporting Items of Systematic Review and Meta-analysis (PRISMA)-compliant literature search was conducted to identify studies describing the use of a percutaneous needle tenotomy in the treatment of idiopathic CTEV. A retrospective case series of patients with idiopathic CTEV treated with percutaneous needle tenotomy over a seven-year period from a single center were also conducted. The patients' demographics, the location of the clubfoot, and the Pirani score were all recorded. An analysis of descriptive statistics was carried out. Continuous data were expressed as mean and SD, whereas categorical variables were expressed as absolute numbers and percentages (%).

The systematic review included eight papers with a total of 1026 feet and a mean age of 10.4 weeks (SD 5.9). There were 47 (0.04%) complications across all studies, with a pooled success rate of 95%. Eleven patients (16 feet) were included in the single-center case study. The patients' initial Pirani score was 4.8 (SD 1.5), with a final score of 0. (SD 0). Four complications occurred in the patient's cohort - one minor bleeding and three recurrences as a result of poor compliance with the post-tenotomy foot abduction brace. In conclusion, the percutaneous Achilles tenotomy of a CTEV foot with a large bore needle is a safe and effective alternative.

## Introduction and background

Congenital talipes equinovarus (CTEV) or pediatric clubfoot is one of the most prevalent congenital lower limb conditions to affect newborns with an incidence of one in 1,000 [1]. It is characterized by cavus and adduction of the forefoot, varus hindfoot, and equinus deformity of the hindfoot. If left untreated, CTEV can lead to long-term deformity and disability [2]. The Ponseti method is recognized as the gold standard treatment for the condition and has been used successfully worldwide [3-5]. The method entails a regime of serial casting with or without an Achilles tenotomy followed by a period of the child in a foot abduction brace (FAB). The rate of necessary Achilles tenotomy has been reported as high as 90% [6]. This is commonly done in an outpatient or theatre environment using a scalpel [7]. However, with the neurovascular bundle being close to the Achilles tendon, complications such as bleeding, nerve injury, and pseudo-aneurysms have been associated with this procedure [8-10].

Minokowitz et al. were the first to describe Achilles tendon needle tenotomy in the literature with the use of a large bore needle as an alternative to a scalpel which is cost-effective, less disruptive for the family, and more comfortable for the child [11]. Although this technique has been reported in a number of literature, most of them have been conducted in Asian countries with no study or case series has been conducted in the United Kingdom [12-18].

The aims of our study were to 1) conduct a systematic review of the literature on outcomes and complications associated with the use of percutaneous needle tenotomy in the treatment of CTEV and 2) report a single-center case series with our outcomes and complications utilizing this technique.

## Review

Systematic review

A study protocol was set out with the study’s objectives, inclusion and exclusion criteria, outcome measures, risk of bias assessment, data abstraction and synthesis. The systematic review was performed using the Preferred Reporting Items of Systematic Review and Meta-analysis (PRISMA) guidelines [19]. It was prospectively registered on PROSPERO: registration number CRD42021256745.

Search Strategy

A literature search was conducted using the following databases: MEDLINE, EMBASE, EMCARE, Cumulative Index of Nursing and Allied Health Literature (CINAHL), Allied and Complementary Medicine Database (AMED) and Cochrane. These databases were provided by the interfaces: OVID and EBSCOhost. The search strategy was run from their inception up to February 2022. For the literature search, a list of relevant mesh terms was developed (Supplementary 1). There were no limits on language, publication date, or journal.

The authors made reasonable efforts to obtain English translations of potentially relevant non-English language studies; where this was not possible, the studies were excluded.

Selection of Studies

After the removal of duplicates and studies not available in English, two reviewers (MD and FE) independently screened the paper title and abstract. The following were the inclusion criteria: 1) a newborn with idiopathic CTEV, 2) treatment with the Ponseti method, and 3) a percutaneous needle tenotomy. Adult CTEV, delayed presentation, syndromic or atypical CTEV, case reports, conference abstracts, systematic reviews, non-clinical (laboratory or biomechanics), and non-human (animal or predictive models) studies were all excluded from the review. Any contention between the choice of studies was arbitrated by a third reviewer (HHC or SS). Reference lists from full-text articles were also screened for additional studies that might be of interest.

Data Extraction and Analysis

Two reviewers (MD and FE) independently extracted data from the selected full-text articles. This includes paper title, authors, year of publication, country of study, study type, sample size, number of feet, mean age in weeks, gender, unilateral or bilateral feet, needle gauge size, procedure setting, the scoring system used (Pirani or Dimeglio), pre- and post-procedure outcomes, complications, need for further intervention and average follow-up. Descriptive statistics were used to summarize the outcome based on pooled raw data.

Risk of Bias Assessment

The risk of bias in each study was assessed using Joanna Briggs Institute (JBI) critical appraisal tool [20]. The studies were assessed using the JBI 10 to 13 screening questionnaire depending on whether they were case series or a randomized controlled trial (RCT).

Case series

A retrospective case series was conducted on newborn babies who received Ponseti treatment for idiopathic CTEV with percutaneous needle Achilles tenotomy at our local institute between 2014 and 2021. Patients with relapsed CTEV, syndromic CTEV, atypical CTEV or received scalpel tenotomy were excluded from the study.

Data were collected on demographics including age, affected foot, Pirani score (pre-cast, pre-tenotomy, post-tenotomy), and the number of serial casts before tenotomy. At our institute, babies with CTEV were seen in the pediatric orthopedic service within seven to 10 days of birth. They were given weekly manipulation, Pirani grading, and serial plaster casting according to the Ponseti method. After casting, the babies were evaluated for any residual fixed equinus deformity. Achilles tenotomy was considered for patients with a midfoot contracture score (MCS) and a hindfoot contracture score (HCS) of more than one.

All parents were informed of the procedure and written consent was taken. The procedure was performed in an outpatient setting. EMLATM 5% anesthetic cream was applied to the Achilles tenotomy site 45 minutes before the procedure. The child was fed by a parent whilst lying in a prone or lateral position. Equinus deformity of the ankle was assessed. The foot was maximally dorsiflexed until the Achilles tendon was taut and easily palpable. One finger breath from the posterior heel crease, a sterile 18-gauge needle was inserted medial to the Achilles tendon. The beveled edge of the needle was moved medially-laterally in a grating motion until the tendon was felt to fully give way. Confirmation of a successful procedure was obtained by achieving at least 10-20 degrees of ankle dorsiflexion. Bleeding in the area was controlled with constant digital pressure for five minutes. An above-knee plaster with 10 degrees dorsiflexion and external rotation was then applied to the affected limb for three weeks before being converted into FOB.

A descriptive statistical analysis was performed using Microsoft Excel software. Continuous data were expressed as mean and standard deviation (SD), whereas categorical variables were expressed as absolute numbers and percentages (%). Prior to the study, our institutional review board was consulted. Formal ethical approval was deemed not necessary because it only included retrospective case notes.

Results

Systematic Review Findings

**Figure 1:**
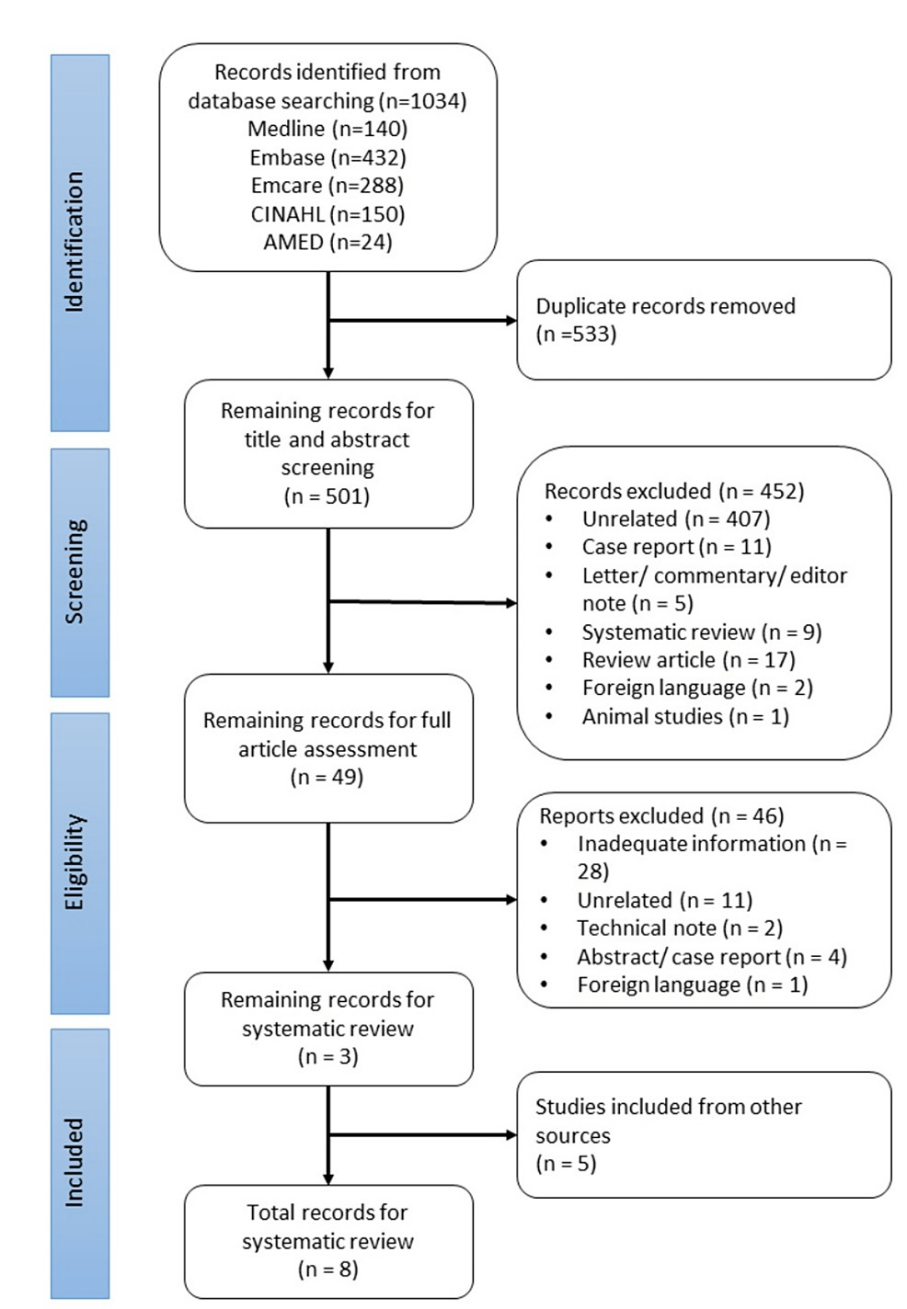
Prisma diagram showing the search strategy utilized for the systematic review

The electronic search strategy yielded a total of 1,034 papers. After removing duplicates, 501 studies were subjected to an abstract screening, with 452 being excluded. The final 49 studies had a full-text review and only two were included in the final review. Six studies were identified through reference searches and included in the review, for a total of eight studies [12-18,21]. Seven studies were prospective cohort studies, and one study was an RCT. Figure 1 shows the results of the literature search as a PRISMA flow chart.

Characteristics of Selected Studies

There were a total of 1,026 feet treated using percutaneous needle Achilles tenotomy. The mean age was 10.4 weeks (SD 5.9). The average follow-up was 5.4 months (SD 1.4 months) however only four of the included studies listed their length of follow-up. The needle sizes used for the tenotomy were usually 16 gauge; however, two studies used either 16- or 18-gauge needles depending on the patient's age [17,18] and Rahman et al. used a 19-gauge needle [15]. All procedures were carried out in an outpatient setting. The characteristics of the selected studies are shown in Table 1.

**Table 1:** Main Characteristics of Eligible Studies Included in the Systematic Review. CS = cohort study; RCT = randomized controlled trial; * = not reported in studies; a = 2 feet did not require tenotomy; b = reported scalpel and needle tenotomy, only needle tenotomy data extracted

Author / Year	Maranho 2010 [21]	Sirsikar 2014 [16]	Rahman 2014 [15]	Choubey^b^ 2015 [13]	Alva 2017 [12]	Evans 2017 [14]	Pandey 2017 [17]	Khan 2018 [18]
Country	Brazil	India	India	India	India	Bangladesh	India	India
Study design	CS	CS	CS	RCT	CS	CS	CS	CS
Population (n=)	39	33	52	*	25	452	37	25
Number of feet	59	49	70	28	45	690	51	34
Mean Age (weeks)	16.70	5.75	17.42	3.75	6.50	*	12.4	*
Gender (Male / Female)	*	20 / 13	32 / 20	*	14 / 11	276 / 176	29 / 8	14 / 11
Unilateral / Bilateral	19 / 20^a^	17 / 16	34 / 18	*	5 / 20	214 / 238	23 / 14	16 / 9
Needle size (Gauge)	16	16	19	16	16	*	16-18	16-18
Mean Follow up length (months)	*	7	4.5	4	*	*	6	*

Outcomes Measures of Needle Tenotomy

Table 2 summarized the pre- and post-tenotomy scoring, recurrence rates and overall success of the procedure of the included studies. Four studies used the Pirani scoring system [22] to assess the severity of the clubfoot of patients pre- and post-tenotomy [12,13,15,17]. The mean pre-tenotomy Pirani score was 5.0 (SD 0.1) with the mean post-tenotomy score being 0.4 (SD 0.1). Sirsikar et al. [16] used the Dimeglio grading system [23] in their study with a mean pre-tenotomy grade of 2.55 and a post-tenotomy grade of 1.05. In all eight papers analyzed, recurrence of clubfoot after tenotomy was observed in 10 cases. In the use of needle tenotomy, the overall success rate was 95% (SD 6.1%) across all investigations.

**Table 2:** Pre- and post-tenotomy scoring, recurrence and overall outcome reported in the eligible studies. * = Information not reported in the studies

Study	Scoring	Pre-tenotomy (Mean)	Post tenotomy (Mean)	Recurrence	Outcome (% of success)
Maranho et al. [21]	Nil	*	*	*	100%
Sirsikar et al. [16]	Nil	*	*	4	90%
Rahman et al. [15]	Pirani	4.90	0.75	3	96%
Choubey et al. [13]	Pirani	5.58	0.31	1	96%
Alva et al. [12]	Pirani	4.90	*	0	100%
Evans et al. [14]	Nil	*	*	*	*
Pandey et al. [17]	Pirani	4.75	0.07	0	100%
Khan et al. [18]	Dimeglio	2.55	1.05	2	84%
				Mean	95%

Complications of Needle Tenotomy in the Selected Studies

The total number of complications in all studies was 47. The most common complication was prolonged bleeding with 21 cases. No study reported cases of neurovascular compromise, infection, or pseudoaneurysm. All the complications are documented in Table 3.

**Table 3:** Complications related to needle tenotomy reported in the eligible studies. * = Complications not reported in studies

Study	Total Complications	Procedural Issues	Incomplete Tenotomy	Abnormal Bleeding	Skin Compromise	Other
Maranho et al. [21]	2	*	*	2	*	*
Sirsikar et al. [16]	5	*	*	*	5	*
Rahman et al. [15]	9	4	3	2	*	*
Choubey et al. [13]	2	*	1	*	1	*
Alva et al. [12]	5	2	*	3	*	*
Evans et al. [14]	11	*	*	11	*	*
Pandey et al. [17]	11	1	2	3	5	*
Khan et al. [18]	2	*	*	*	*	2

Risk of Bias

Table 4 displays the results of the JBI risk of bias assessment for each paper. Of the eight studies included, six were shown to be low risk, one as moderate risk and one as high risk.

**Table 4:** Joanna Briggs Institute risk of bias assessment. NA = Not applicable, ✔= Yes, ✘= No

(a) Joanna Briggs Institute risk of bias assessment for case series																									
	Q1		Q2		Q3		Q4			Q5		Q6		Q7		Q8			Q9		Q10		%Yes		Risk
Maranho et al.	✔		✔		NA		✔			✔		✔		✔		✘			✔		✔		80		Low
Sirsikar et al.	✔		✔		NA		✔			✔		✔		✔		✘			✔		✔		80		Low
Rahman et al.	✘		✔		NA		✔			✔		✔		✔		✔			✔		✔		80		Low
Alva et al.	✔		✔		NA		✔			✔		✔		✔		✔			✔		✔		90		Low
Evans et al.	✘		✘		NA		✔			✔		✘		✘		✘			✔		✔		40		High
Pandey et al.	✔		✔		NA		✔			✔		✔		✔		✘			✔		✔		80		Low
Khan et al.	✔		✘		NA		✔			✔		✔		✔		✔			✔		✔		80		Low
(b) Joanna Briggs Institute risk of bias assessment for randomized controlled trials																									
	Q1	Q2		Q3		Q4		Q5	Q6		Q7		Q8		Q9		Q10	Q11		Q12		Q13		%Yes	Risk
Choubey et al.	✔	NA		✔		NA		NA	NA		✔		✔		✔		✘	✔		✔		✔		62	Moderate

Case Series Outcomes

Twenty-six patients were identified in the records as having undergone treatment for clubfoot. Of these, 15 were excluded from the report; five had undergone a knife tenotomy, three had spina bifida or atypical neurology, and seven did not require a tenotomy post successful serial casting. Of the 11 patients included in the study, seven were male and four females. Five patients had bilateral involvement and six patients were unilateral, resulting in 16 feet in total. The average initial Pirani score was 4.8 (SD 1.5) with a mean number of serial casts of five (SD 1). The average mean pre-needle tenotomy score was 1.2 (SD 0.7) and the average post-tenotomy was 0 (SD 0). Till date, the shortest follow-up patient was six months while the longest follow-up was seven years. The results are summarized in Table 5.

**Table 5:** Demographics of local institute case series. SD = Standard deviation

Affected foot	Bilateral (n = 5)	45%
	Unilateral (n = 6)	55%
Mean Initial Pirani Score		4.8, SD 1.5
Mean Number of Serial Casts		5, SD 1
Mean Pre-tenotomy Pirani Score		1.2, SD 0.7
Mean Post-tenotomy Follow-up Score		0, SD 0

There were four complications recorded for these patients. One patient had post-tenotomy bleeding with blood seeping through the cast, mandating a cast change one-week post-procedure. The cast was removed followed by constant digital pressure applied for five minutes, and the above-knee cast was reapplied without any further problems. Three patients suffered a relapse of their CTEV due to poor compliance with boots and bars. One patient was required to repeat serial casting with tenotomy, one patient required tibialis anterior transfer at an older age, and another one is currently under monitoring.

Discussion

The Ponseti method's success has been well established in the literature since its inception [5,24-26]. Despite the fact that the method was first described with an Achilles tenotomy performed with a scalpel [3], considerable complications have been reported with this method [8-10]. This is owing to the posterior neurovascular bundle's proximity to the Achilles tendon, which is posteromedial to the tendon. This systematic review and single-center case series have shown that the use of the percutaneous needle is a viable alternative in performing an Achilles tenotomy. To our knowledge, this is the first systematic review on the topic. Although Pandey et al. [17] performed a literature search in their work, they did not present a systematic methodology.

Studies have shown there are variations in the clubfoot population with a poorly developed anterior neurovascular bundle and a dominant posterior bundle supplying the foot [27,28]. This emphasizes the importance of preserving the posterior bundle intact while undergoing an Achilles tenotomy for CTEV. Approaches such as a mini-open technique have been described in the literature to ensure the protection of the bundle [7]. However, these techniques often require a theatre setting, can be distressing for the parents of the child and is more resource intensive. According to our findings, percutaneous needle tenotomy can be performed safely and effectively in an outpatient setting with adequate parental counseling.

Choubney et al. performed an RCT comparing percutaneous scalpel tenotomy to needle tenotomy [13]. They found no statistically significant difference in post-treatment Pirani score between the needle and scalpel tenotomy (blade score 0.26 versus needle score 0.31, p>0.05). However, due to the need for parental participation and consent, the study was unable to be blinded, which is understandable. Maranho et al., on the other hand, used ultrasonography to evaluate the completion of their percutaneous needle Achilles tenotomy post-procedure. They found that in most cases, the tendon was completely sectioned [21]. Overall, we have seen a positive outcome with needle tenotomy in the included studies, with a successful outcome in 95% of the patients.

There was no evidence of neurovascular compromise, pseudoaneurysm, or infection in our systematic review and case series. Prolonged bleeding was the most common immediate postoperative complication, with one case reported in our series; however, all cases were managed with prolonged digital pressure over the bleeding site. Three relapses were reported in our study, all of which were due to poor compliance with the FAB rather than the needle tenotomy procedure itself. A repeat tenotomy was required in one patient, while an anterior tibialis transfer was performed in the other. Overall, needle tenotomy has a low relapse rate, with most relapses in the studies attributed to poor FAB compliance rather than the procedure itself.

There are limitations to our study. The studies included in this review, with the exception of one RCT, are mostly of level four evidence, including our own case series. As a result, this review did not include a meta-analysis since no studies included comparators in their design. The sample sizes for each study, including our own, were also relatively modest. This is due to the studies all stemming from a single center, as well as the low incidence of CTEV in the general population, with Evans et al. [14] having the most patients with 452 in a two-year period. All the studies in the analysis had short follow-up periods, with the average being seven months. This may not be enough time to record any relapses that may have occurred.

In our case series, the follow-up duration spans from six months to seven years. Finally, this study and our case series focused solely on idiopathic clubfoot. This is because both syndromic and atypical CTEV is known to have significant recurrence rates and treatment techniques differ greatly [6,29,30].

## Conclusions

An Achilles tenotomy is required in at least 90% of CTEV cases to correct the final equinus deformity. Using a large bore needle to perform an Achilles tenotomy in CTEV patients is an effective and safe alternative to using a scalpel, according to our review and case series. Complications from percutaneous needle tenotomy are extremely rare, and the clinical outcome is comparable to traditional scalpel tenotomy. However, higher powered, multi-centered studies are required to back this up.

## Appendices

Search Strategy

Interface: EBSCOhost Research Databases (CINAHL)

Search date 27/5/21

1. (MH "Clubfoot")

2. TI ( Clubfoot or clubfeet ) OR AB ( Clubfoot or clubfeet )

3. TI CTEV OR AB CTEV

4. TI congenital talipes equinovarus OR AB congenital talipes equinovarus

5. S1 OR S2 OR S OR S4

6. "percutaneous needle tenotomy"

7. TI percutaneous needle tenotomy OR AB percutaneous needle tenotomy

8. "tenotomy"

9. TI tenotomy OR AB tenotomy

10. S6 OR S7 OR S8 OR S9

11. S5 AND S10

Interface: OVID Medline, Embase, Emcare, AMED

Search date 27/5/21

1. exp Clubfoot/

2. (clubfoot or clubfeet).ti,ab.

3. congenital talipes equinovarus.mp.

4. CTEV.mp.

5. 1 or 2 or 3 or 4

6. percutaneous needle tenotomy.mp.

7. "Needle tenotomy".kw.

8. needle tenotomy.mp.

9. Tenotomy/

10. 6 or 7 or 8 or 9

11. 5 and 10

12. exp Clubfoot/

13. (clubfoot or clubfeet).ti,ab.

14. congenital talipes equinovarus.mp.

15. CTEV.mp.

16. 12 or 13 or 14 or 15

17. percutaneous needle tenotomy.mp.

18. Needle tenotomy.mp.

19. Tenotomy/

20. 17 or 18 or 19

21. 16 and 20

22. remove duplicates from 21

Interface: Cochrane CENTRAL

Search Date: 27/05/2021

1.. MeSH descriptor: [Clubfoot] explode all trees

2. (clubfoot or clubfeet):ti,ab,kw

3. CTEV

4.(congenital talipes equinovarus):ti,ab,kw

5.{or #1-#4}

6. (percutaneous needle tenotomy):ti,ab,kw

7. (needle tenotomy):ti,ab,kw

8. MeSH descriptor: [Tenotomy] explode all trees

9. {or #6-#8}

10. #9 and #5
